# In Vitro Anti-proliferative Activity and Mechanism of Action of *Anemone nemorosa*

**DOI:** 10.3390/ijms20051217

**Published:** 2019-03-11

**Authors:** Bresler Swanepoel, Luanne Venables, Octavian Tudorel Olaru, George Mihai Nitulescu, Maryna van de Venter

**Affiliations:** 1 Department of Biochemistry and Microbiology, PO Box 77000, Nelson Mandela University, Port Elizabeth 6031, South Africa; s211129399@mandela.ac.za (B.S.); s204004039@mandela.ac.za (L.V.); Maryna.VanDeVenter@mandela.ac.za (M.v.d.V.); 2 Faculty of Pharmacy, “Carol Davila” University of Medicine and Pharmacy, Traian Vuia 6, Bucharest 020956, Romania; octavian.olaru@umfcd.ro

**Keywords:** *A. nemorosa*, cytotoxicity, apoptosis, HeLa

## Abstract

*Anemone nemorosa* is part of the Ranunculaceae genus *Anemone* (order Ranunculales) which comprises more than 150 species. Various parts of the plant have been used for the treatment of numerous medical conditions such as headaches, tertian agues, rheumatic gout, leprosy, lethargy, eye inflammation as well as malignant and corroding ulcers. The *Anemone* plants have been found to contain various medicinal compounds with anti-cancer, immunomodulatory, anti-inflammatory, anti-oxidant and anti-microbial activities. To date there has been no reported evidence of its use in the treatment of cancer. However, due to the reported abundance of saponins which usually exert anti-cancer activity via cell cycle arrest and the induction of apoptosis, we investigated the mode of cell death induced by an aqueous *A. nemorosa* extract by using HeLa cervical cancer cells. Cisplatin was used as a positive control. With a 50% inhibitory concentration (IC_50_) of 20.33 ± 2.480 µg/mL, treatment with *A. nemorosa* yielded a delay in the early mitosis phase of the cell cycle. Apoptosis was confirmed through fluorescent staining with annexin V-FITC. Apoptosis was more evident with *A. nemorosa* treatment compared to the positive control after 24 and 48 h. Tetramethylrhodamine ethyl ester staining showed a decrease in mitochondrial membrane potential at 24 and 48 h. The results obtained imply that *A. nemorosa* may have potential anti-proliferative properties.

## 1. Introduction

Normal physiological processes are maintained through a homeostatic balance between two very critical parts of the normal development and maturation cycle namely cell proliferation and cell death. Any alterations to this homeostatic balance can lead to diseases such as AIDS, diabetes, neurodegenerative diseases, and cancer.

Apoptosis is a cellular suicide mechanism that is regulated by a family of cysteine proteases otherwise known as caspases [1]. Apoptosis is characterized by: a reduction in mitochondrial transmembrane potential, intracellular acidification, cell shrinkage, DNA fragmentation and condensation, production of reactive oxygen species, externalization of phosphatidylserine and selective proteolysis of a subset of cellular proteins [2]. It is sub-classified into two non-exclusive types of death pathways namely the extrinsic (receptor-mediated) and intrinsic (mitochondria-mediated) pathway [3]. Cancer cells have the ability to resist apoptotic insults by means of various mechanisms and therefore a thorough understanding of these mechanisms is imperative to unravel the secret to designing more effective and targeted therapeutic strategies [4].

*Anemone* is a genus of more than 150 species of flowering plants that are native to the temperate zones of both the Northern and Southern hemispheres. Of the 150 species, more than 50 are used in various traditional medical systems. In China alone, 53 species, 9 subspecies, and 36 varieties are found in most provinces of which at least 38 species/varieties have ethnopharmacological uses [5]. Observed pharmacological activities include anti-cancer, anti-microbial, anti-inflammatory, sedative, and analgesic activities as well as anti-convulsant and anti-histamine effects. Various parts of *A. nemorosa* have been used for the treatment of numerous medical conditions such as headaches, tertian agues, rheumatic gout, leprosy, lethargy, eye inflammation, malignant ulcers and as an antimicrobial, antifungal, diuretic, and abortive agent [5,6].

**Figure 1:**
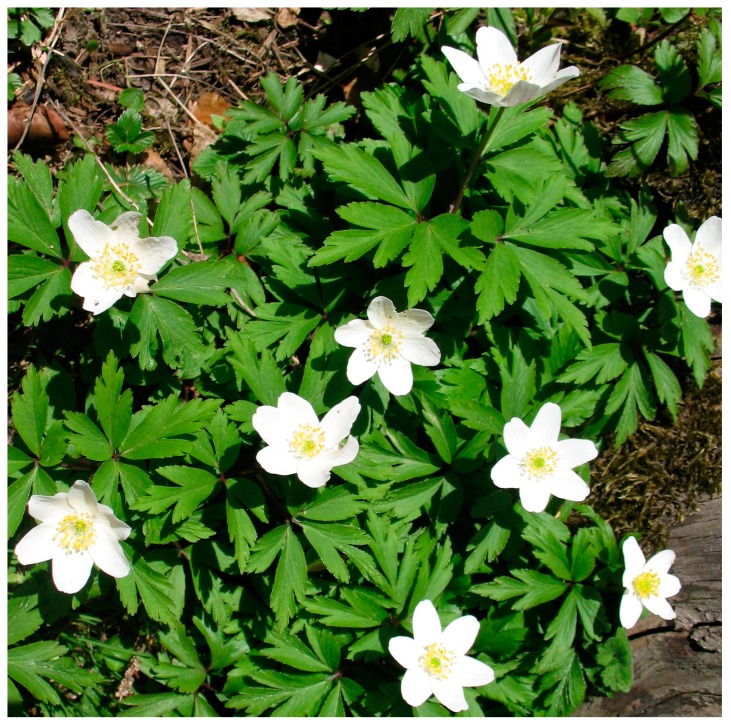
*Anemone nemorosa* before harvesting.

Although *A. nemorosa* is not used traditionally for anti-cancer treatment, reported evidence shows the presence of compounds responsible for anti-cancer activity such as triterpenoids and saponins [7]. For this reason, we investigated the mode of cell death caused by an aqueous extract from *A. nemorosa* (Figure 1) on HeLa cervical cancer cells. The aqueous extract was selected based on a preliminary anti-proliferative screening of ethanolic, hydroethanolic, and aqueous extracts.

## 2. Results

### 2.1. Cytotoxicity

**Figure 2:**
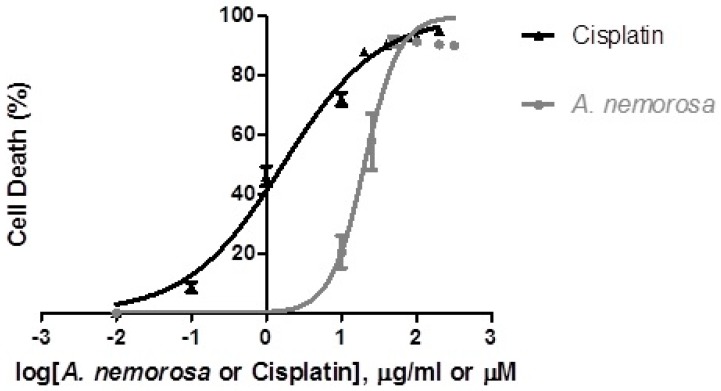
Cytotoxic effect of *A. nemorosa* on HeLa cancer cells after 48 h of exposure. Cell viability was determined using the Hoechst 33342/propidium iodide (PI) staining method. Error bars indicate SD of four replicate values of three individual experiments.

Cancer cells accumulate multiple mutations in genes that regulate the cell cycle. Certain mutations occur more frequently than others and the sensitivity of cancer cells to anti-cancer treatment is influenced by the specific mutations in that cancer. The cytotoxic effect of *A. nemorosa* was determined by Hoechst 33342/propidium iodide (PI) dual staining for HeLa (Figure 2), MeWo, and HepG2 cancer cells. The 50% inhibitory concentration (IC_50_) values obtained were 20.33 ± 2.480 µg/mL, >200 µg/mL and 27.66 ± 12.27 µg/mL, respectively (data not shown for MeWo and HepG2 cells). Following these results, all subsequent experiments were performed on HeLa cells using the determined IC_10_, IC_25_, IC_50_, and IC_75_ values (Table 1).

### 2.2. Cell Cycle Analysis

**Figure 3:**
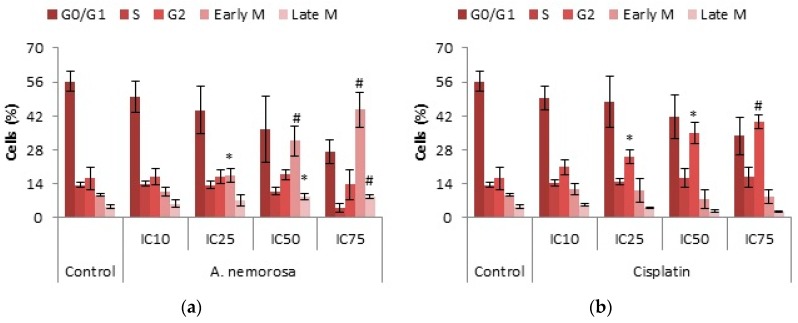
Cell Cycle Analysis of HeLa cells after 24 h of treatment with *A. nemoroasa* (**a**) and cisplatin (**b**). Cell cycle analysis was determined by the NucRed Live 647 staining method. Error bars indicate SD of four replicate values of three individual experiments. Significance was determined using the two-tailed Student *t*-test: * *p* < 0.05 and ^#^ *p* < 0.005 compared to control.

DNA cell cycle analysis was performed to determine arrest of cells in a certain phase of the cell cycle. HeLa cells were exposed to *A. nemorosa* at its respective IC_10_, IC_25_, IC_50_, and IC_75_ concentrations for 24 and 48 h. As shown, treatment with cisplatin arrested cells in the G2 phase whereas with *A. nemorosa* arrest occurred in the early M phase (Figure 3). The same was seen after 48 h for treatment with cisplatin but early M phase arrest for *A. nemorosa* was not as pronounced (Figure S1).

### 2.3. Histone H3 Phosphorylation

Phosphorylation of Histone H3 at Ser10 is believed to be a marker for cells entering mitosis. Increased levels of phosphorylated histone H3 confirms cell cycle arrest in the M phase. Immunofluorescence staining with phospho-H3 (ser10) antibody was performed after 24 and 48 h of extract treatment. No significant increase in the percentage of phosphorylated H3 was observed after 24 or 48 h, except for the IC_75_ treatment of *A. nemorosa* (Table 2). It was also evident that there was a decrease in phosphorylated histone H3 after 48 h which could support the low percentage of cells in the early M phase of the cell cycle analysis after 48 h.

### 2.4. Micronucleus Assay

**Figure 4:**
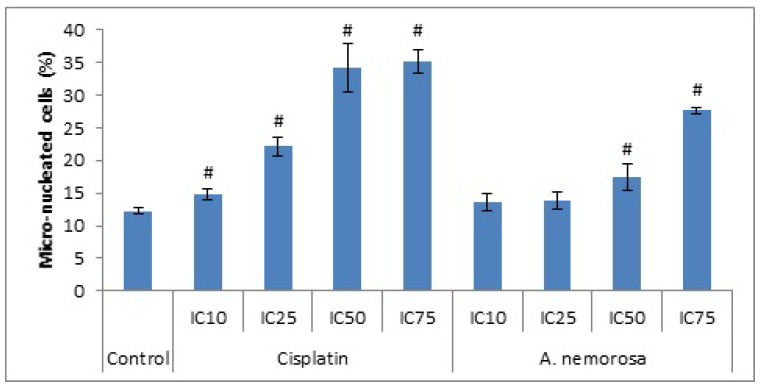
Assessment of genotoxicity in HeLa cells after 48 h using the NucRed Live 647 staining method. Error bars indicate SD of four replicate values of three individual experiments. Significance was determined using the two-tailed Student *t*-test: ^#^ *p* < 0.005 compared to control.

‘Micronuclei’ refers to small nuclei that are formed whenever a chromosome or a fragment of a chromosome is not incorporated into one of the daughter nuclei during cell division [8]. It is usually a sign of genotoxic events and chromosomal instability. It is therefore believed that the formation of micronuclei is associated with M phase arrest and more specifically mitotic catastrophe [9]. A significant increase in the formation of micronuclei was seen following 24 h (Figure S2) and 48 h of exposure to *A. nemorosa* at its IC_50_ and IC_75_ (Figure 4).

### 2.5. Phosphatidylserine (PS) Translocation

**Figure 5:**
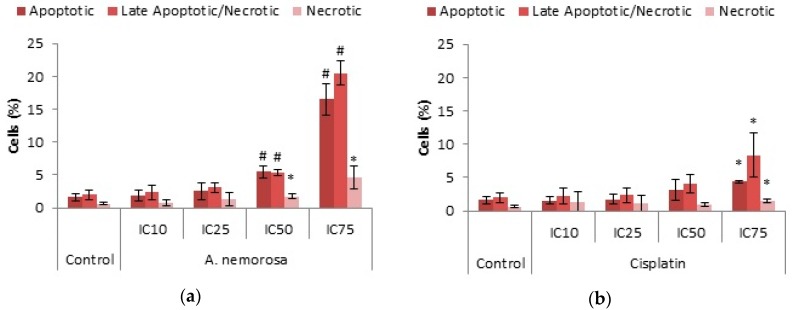
Analysis of phosphatidylserine (PS) translocation in HeLa cells using Annexin V-FITC and PI dual staining after 48 h of treatment with *A. nemoroasa* (**a**) and cisplatin (**b**). Results displayed as percentage positively stained cells. Error bars indicate SD of four replicate values of three individual experiments. Significance was determined using the two-tailed Student *t*-test: * *p* < 0.05 and ^#^ *p* < 0.005 compared to control.

PS translocation is considered an early feature of apoptosis due to the loss of membrane integrity upon induction of apoptosis. Annexin V, is a 35–36 kDa calcium-dependent phospholipid-binding protein that is capable of binding to PS with high affinity [10]. The presence of PS was determined by Annexin V-FITC and PI staining after 24 (Figure S3) and 48 h. Results recorded indicated that more PS was present after 48 h of treatment (Figure 5). Significant increases were seen for all treatments of *A. nemorosa* in a dose dependent manner with the greatest increase in the number of apoptotic cells recorded for the IC_75_ treatment.

### 2.6. Mitochondrial Membrane Potential (MMP) Analysis

**Figure 6:**
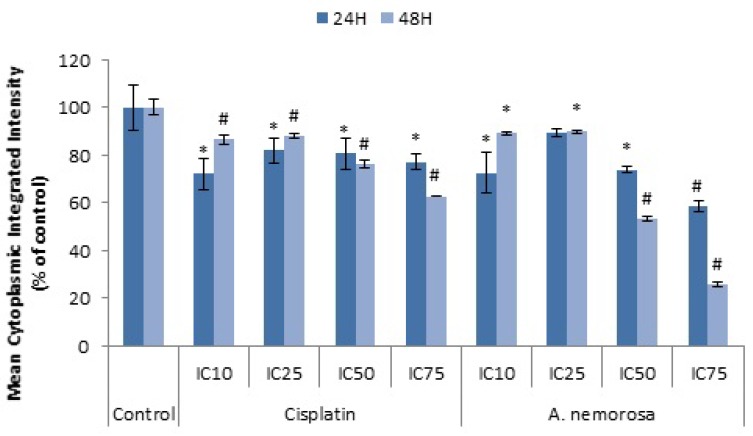
Changes in mitochondrial membrane potential (MMP) after 24 and 48 h of exposure to cisplatin and *A. nemorosa*. Results displayed as mean cytoplasmic integrated intensity. Error bars indicate SD of four replicate values of three individual experiments. Significance was determined using the two-tailed Student t-test: * *p* < 0.05 and ^#^ *p* < 0.005 compared to control.

The onset of the intrinsic mode of apoptosis is believed to be indicated by the loss of mitochondrial membrane potential and the subsequent release of pro-apoptotic proteins. The mitochondrial membrane potential was measured by using a lipophilic cationic dye, tetramethylrhodamine ethyl ester (TMRE), which reversibly accumulates in the mitochondrial matrix in a voltage dependent manner. During apoptosis the ability of TMRE to accumulate inside the mitochondria is hampered and the dye becomes evenly distributed throughout the cytosol. This leads to an overall drop in the fluorescence intensity which can then be quantified by fluorescence microscopy (Figure 6) [11]. A significant decrease in the mean cytoplasmic integrated intensity was seen after 24 and 48 h of exposure to *A. nemorosa* compared to the control (Figure 5). This suggests depolarization of the mitochondrial membrane and therefore the involvement of mitochondria in the onset of apoptosis.

### 2.7. Caspase 8 and 3 Activation

Caspase 8 and 3 are the two main caspases involved in the initiation and execution of apoptosis, respectively. Activation of caspase 8 and 3 was determined after 24 and 48 h by immunofluorescence staining with antibodies against activated/cleaved caspase 8 and 3. An increase in the mean cell integrated intensity values, when compared to the control, indicates the presence of cleaved, or activated, caspase 8 or 3. Treatments with *A. nemorosa* indicated significant dose dependent increases in the mean cell integrated intensity for both caspase 8 and 3 after 24 and 48 h of exposure (Table 3 and Table 4).

### 2.8. Reactive Oxygen Species (ROS) Production

**Figure 7:**
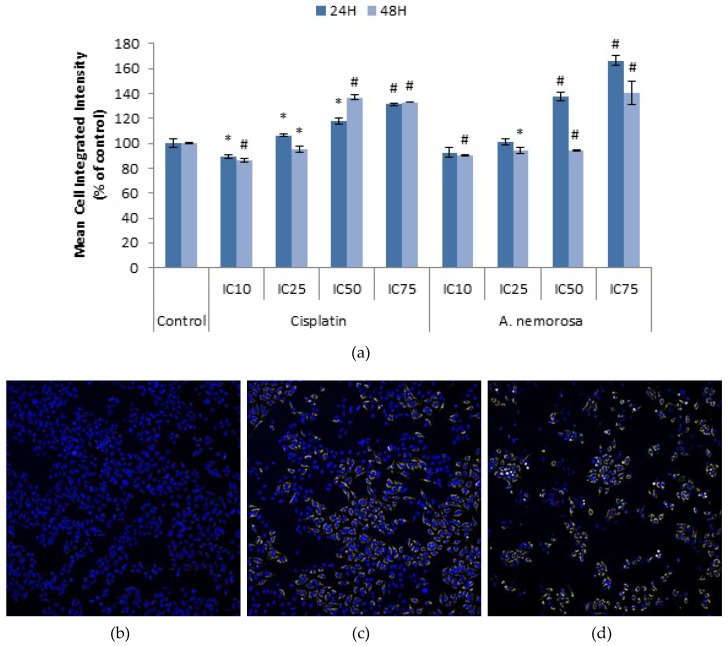
Changes in levels of reactive oxygen species for measurement of oxidative stress in HeLa cells after 24 and 48 h of exposure to cisplatin and *A. nemorosa* (**a**). Results displayed as mean cell integrated intensity. Micrographs (10× magnification) indicating positive staining for reactive oxygen species in HeLa cells after 24 h of treatment compared to control (**b**), cisplatin (**c**), and *A. nemorosa* (**d**). Cells were stained with CellRox Orange and Hoechst 33342. Nuclei: blue; reactive oxygen species: yellow. Error bars indicate SD of four replicate values of three individual experiments. Significance was determined using the two-tailed Student *t*-test: * *p* < 0.05 and ^#^ *p* < 0.005 compared to control.

The levels of reactive oxygen species in cells, under normal physiological conditions, are controlled by the balance between anti-oxidants and free radicals. Excessive production of reactive oxygen species can induce lipid peroxidation, depletion of sulfhydryl groups, altered signal transduction pathways, altered calcium homeostasis and DNA damage, which could lead to the induction of apoptosis [12]. CellRox orange is a dye that indicates the presence of reactive oxygen species. An increase in the mean cell integrated intensity indicates an increase in the levels of reactive oxygen species. HeLa cells treated with *A. nemorosa* were stained with CellRox orange and images were acquired (Figure 6). Exposure to *A. nemorosa* indicated more significant increases, compared to the control, in the mean cell integrated intensity after 24 h as opposed to 48 h (Figure 7).

### 2.9. Autophagy Induction

**Figure 8:**
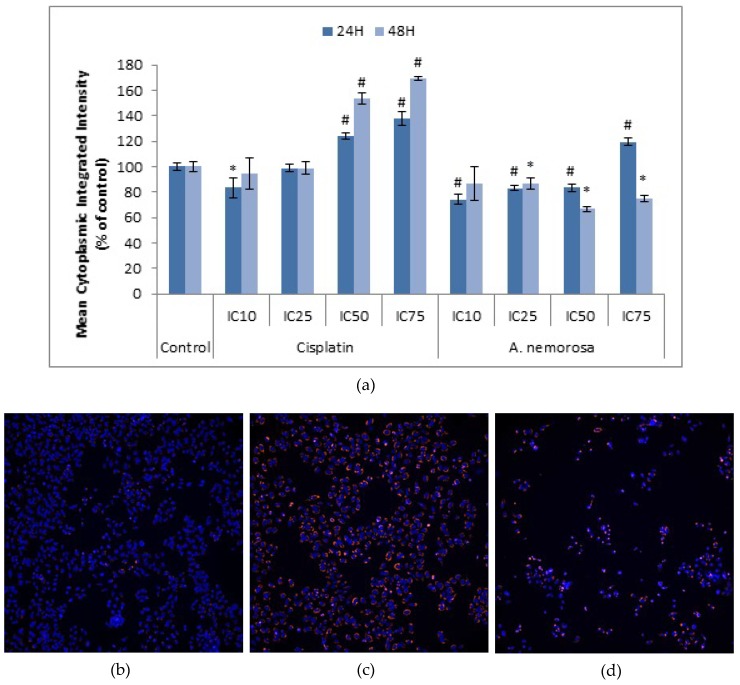
Changes in levels of acidic organelles after 24 and 48 h of exposure to cisplatin and *A. nemorosa*. Results displayed as mean cytoplasmic integrated intensity (**a**). Micrographs (10× magnification) indicating positive staining for acidic organelles in HeLa cells after 48 h of treatment compared to control (**b**), cisplatin (**c**), and *A. nemorosa* (**d**). Cells were stained with Lysotracker Red and Hoechst 33342. Nuclei: blue; acidic organelles: orange. Error bars indicate SD of four replicate values of three individual experiments. Significance was determined using the two-tailed Student *t*-test: * *p* < 0.05 and # *p* < 0.005 compared to control.

Autophagy is a catabolic process that is regulated by the mammalian target of rapamycin (mTOR) kinase. It is characterized by its homeostatic role in the autophagosomic-lysosomal degradation of: (1) bulk cytoplasmic contents, (2) abnormal protein aggregates and (3) excess or damaged organelles. This role mainly reflects on its pro-survival function but several studies have suggested a role in cell death as monitored by the accumulation of LC3-II in cells in which apoptotic signaling has been perturbed [13]. Lysotracker™ Red is an acidotrophic dye that indicates the presence of late autophagic vesicles and autophagy activation. An increase in the mean cytoplasmic integrated intensity indicates an increase in the number of acidic organelles such as late autophagic vesicles. HeLa cells treated with *A. nemorosa* were stained with Lysotracker™ Deep Red and images were acquired (Figure 8). Significant decreases in LTR staining were evident for treatment with *A. nemorosa* as opposed to significant increases observed for cisplatin treatment after both 24 and 48 h compared to the control (Figure 8).

## 3. Discussion

To date there has been no evidence of the use of *A. nemorosa* for the treatment of cancer. Studies involving *A. nemorosa* mainly consist of anti-microbial activity against known hospital and fish pathogens such as *E. coli* and *Vibrio anguillarum*, respectively [14,15]. However, the isolation of numerous phytochemical compounds from other species of the *Anemone* genus has provided sufficient reasons as to why *A. nemorosa* could be used for cancer treatment.

Raddeanin A is a pentacyclic triterpenoid saponin, isolated from *A. raddeana*, and induces apoptosis in multiple cell lines by means of increased Bax expression, reduced Bcl-2 and Survivin expression and the activation of caspases 3, 8 and 9 in gastric cancer cells [5]. Whilst isolated triterpenoid saponins from *A. flaccida*, hedera saponin StI4a, hedera saponin St-J, anhuienoside E, hedera saponin B and flaccidoside II, have been shown to induce apoptosis in HeLa cervical cancer cells [7].

Dose-response assays (Figure 1) were performed by means of the Hoechst 33342/PI dual staining method and results indicated that *A. nemorosa* inhibits proliferation of HeLa cells in a dose dependent manner. For a crude extract to be considered promising for systemic application, the IC_50_ should be less than 100 µg/mL in vitro [16]. The IC_50_ of *A. nemorosa* was determined to be 20.33 ± 2.480 µg/mL.

One of the basic characteristics of cancer cells is their ability to proliferate uncontrollably. Therefore, DNA cell cycle analysis remains an important aspect in the search for new and improved chemotherapeutic agents. Uncontrollable proliferation can be ascribed to the alteration of the various cell cycle checkpoints [17]. Extracts, such as *A. nemorosa*, with cytotoxic activity may counteract these alterations by activating cell cycle arrest through the damage to the mitotic spindle or by affecting the signaling pathways that regulate proliferation [18]. *A. nemorosa* was shown to dose dependently arrest HeLa cells in the early M phase of the cell cycle after 24 h of exposure whereas cisplatin arrested cells in the G2 phase (Figure 3). The exact mechanism of how *A. nemorosa* and cisplatin cause arrest in the early M and G2 phase, respectively, cannot be deduced from the NucRed staining and more than one possibility exists. There are two parallel cascades that ultimately serve to inactivate the CyclinB1-CDK1 complex subsequently blocking entry into mitosis. The first cascade occurs quite rapidly where Chk1 phosphorylates Cdc25C preventing it from activating CDK1, leading to G2 arrest. The slower second parallel cascade involves the phosphorylation of p53 by means of activating CKI’s (such as p21) which inactivate the CyclinB1-CDK1 complex. Mitotic arrest is mainly due to the disruption of the formation of the mitotic spindle resulting in mitotic catastrophe [19].

Early M phase arrest by *A. nemorosa* was confirmed through staining with phospho-H3 (ser10) antibody as the phosphorylation of histone H3 is believed to be a marker for cells entering into mitosis [20]. Treatment with the IC_75_ value of extract showed a significant increase in phosphorylated histone H3 after 24 h (Table 2). Cisplatin treatments indicated a dose dependent decrease in phosphorylated histone H3 which supports the G2 phase arrest obtained during cell cycle analysis. Treatment with staurosporine has also showed to inhibit histone H3 phosphorylation leading to G2 phase arrest [20]. Studies done by Hans and Dimitrov (2001) showed that during cell division, the phosphorylation of histone H3 is initiated at different phases. However, metaphase chromosomes have been found to always be the most heavily phosphorylatedand suggests a possible role for histone H3 in the passage of cells from metaphase to anaphase [21]. The low level of phosphorylation seen in this study suggests that HeLa cells would then be mitotically arrested in metaphase which is relatively early in the process of mitosis supporting the early M phase arrest obtained for the cell cycle analysis.

Mitotic catastrophe is a regulated process that uses anti-proliferative measures such as apoptosis, necrosis and senescence during defective or failed mitosis in order to prevent proliferation of cells. Mitotic catastrophe is characterized by the formation of giant multinucleated cells and also micronucleated cells, the former is due to clusters of missegregated uncondensed chromosomes, whereas the latter is as a result of lagging chromosomes or chromosome fragments, during anaphase, which are not incorporated into the daughter nuclei during telophase [22]. Cell death through mitotic catastrophe can manifest in a caspase-dependent or -independent manner. The caspase-dependent manner involves the release of cytochrome c following mitochondrial membrane depolarization and oligomerization of Bax/Bak. Caspase-independent cell death on the other hand can occur through a sudden Ca^2+^ overload, oxidative stress and mitochondrial permeability transition facilitated by the permeability transition pore complex which acts as a bridge between the inner and outer mitochondrial membranes [9]. After 48 h of treatment with *A. nemorosa* significant increases were seen in the formation of micro-nucleated cells which suggests the possible onset of mitotic catastrophe (Figure 3).

Phosphatidylserine translocation is believed to be an early feature of apoptosis due to the loss of membrane asymmetry upon induction of apoptosis. During apoptosis the membrane phospholipid PS is translocated from the inner to the outer leaflet of the plasma membrane. During late necrosis, the membrane integrity becomes compromised and Annexin V may enter the cell and bind to PS in the inner leaflet. Therefore, staining of cells with Annexin V is commonly used in conjunction with PI. Non-apoptotic or healthy cells would stain negative for both Annexin V and PI whereas cells undergoing early apoptosis would be positive for Annexin V but negative for PI. Late apoptotic/early necrotic cells would stain positive for both Annexin V and PI, whereas necrotic cells would only be positive for PI [10]. Treatment with *A. nemorosa* after 48 h showed a dose dependent increase in apoptotic, late apoptotic/necrotic and necrotic cells. The most significant increases were seen for percentage of apoptotic and late apoptotic/necrotic cells for both the IC_50_ and IC_75_ treatments (Figure 5). Cells undergoing necrosis can also stain positive for both Annexin V and PI and therefore it cannot be determined whether or not the cells categorized as late apoptotic/necrotic are actually apoptotic or necrotic, respectively.

Mitochondrial membrane potential plays a key role in the generation of ATP through the respiratory chain and opening of the mitochondrial permeability transition pore would lead to a collapse of this potential and the subsequent release of cytochrome c (Cyt-*c*), Smac/DIABLO, Apoptosis Inducing Factor (AIF) and endonuclease G (Endo G) which all play a role in the initiation of apoptosis either through activation of caspases or leading to DNA fragmentation [23,24,25]. Treatment with *A. nemorosa* for 24 and 48 h indicated a decrease in the mean cytoplasmic integrated intensity of TMRE (Figure 6) and therefore suggests the involvement of the mitochondria in the onset of apoptosis by *A. nemorosa*.

Caspases are proteases that contain a cysteine in their active site which cleave in the C-terminal region of aspartate residues and are synthesized as inactive zymogens. Initiator and effector caspases are two groups of caspases involved in cell death. Initiator caspases contain long pro-domains and form aggregates in response to scaffolding co-factors leading to their auto-activation [26]. In contrast, effector caspases are not capable of auto-activation but become activated through cleavage by initiator caspases. Caspase 8 is an initiator caspase whose activation is promoted by CD95 (Fas/APO-1) and tumor necrosis factor receptor 1 (TNFR1). The active fragments, p18 and p10, of caspase 8 are released during its activation. The activated caspase 8 cleaves and activates effector caspase-1, -3, -6, and -7. Caspase 3 is ultimately responsible for the morphological changes, such as DNA fragmentation and cell shrinkage associated with apoptosis [27]. The activation of both caspase 8 and caspase 3 without the depolarization of the mitochondrial membrane potential would therefore suggest that the extrinsic pathway plays a role in the execution of apoptosis. Similarly the activation of caspase 3, preceded by mitochondrial membrane depolarization would suggest a role of the intrinsic pathway in the execution of apoptosis [28]. Significant increases in the mean cell integrated intensities of both caspase 8 and 3 after 24 and 48 h, respectively, was evident in a dose dependent manner (Table 3 and Table 4). Thus it can be deduced that the induction of apoptosis by *A. nemorosa* involves both the extrinsic and intrinsic pathway. Caspase-dependent apoptosis preceded by mitochondrial membrane depolarization is often associated with increased reactive oxygen species generation. ROS are known to interact with mitochondrial permeability transition complex proteins leading to a significant impact on mitochondrial anion fluxes. The release of cytochrome *c* also leads to further ROS increases due to a disrupted electron transport chain [29]. Treatment with *A. nemorosa* significantly increased ROS levels as was evident in the increase in the mean cell integrated intensity (Figure 6). It can therefore be said that ROS contributes to the induction of apoptosis by *A. nemorosa*.

Apoptosis, mitotic catastrophe, autophagy, and regulated necrosis constitute the four major forms of programmed cell death. Cell death is hardly ever due to single pathway activation but rather the result of cross-talk between multiple pathways of which most of the key players participate in more than one signaling cascade. Autophagy is often observed to occur before apoptosis induction, essentially inhibiting it, and is deemed to be a rescue mechanism in which a cell adapts to stress. However, the induction of apoptosis can lead to the cleavage of autophagy-related genes such as ATG3, BECN1, or AMBRA1 which in turn leads to the inhibition of autophagy [22]. Therefore, the negative or decreased staining observed for treatment with *A. nemorosa* (Figure 7) may be attributed to the absence of autophagy, possibly through inhibition, or it may favor apoptosis induction by tipping the cell survival: cell death balance [30].

## 4. Materials and Methods

### 4.1. Reagents

HeLa cervical cancer cells were purchased from Highveld Biological, Johannesburg, South Africa. RPMI 1640 cell culture medium and fetal bovine serum was purchased from GE Healthcare Life Sciences (Logan, UT, USA). Trypsin-EDTA, Dulbecco’s phosphate buffered saline (DPBS) with Ca^2+^ and Mg^2+^ and Dulbecco’s phosphate buffered saline (DPBS) without Ca^2+^ and Mg^2+^ were purchased from Lonza (Wakersville, MA, USA). Trypan blue, bisBenzamide H 33,342 trihydrochloride (Hoechst 33342), cisplatin, penicillin/streptomycin and bovine serum albumin fraction V were purchased from Sigma-Aldrich (St. Louis, MI, USA). NucRed^TM^ Live 647, CellRox^®^ Orange reagent, Lysotracker™ Deep Red and Tetramethylrhodamine ethyl ester (TMRE) were purchased from Molecular Probes^®^-Life Technologies-Thermo Fisher Scientific (Logan, UT, USA). Annexin V-FITC/PI kit was purchased from MACS Miltenyi Biotec (Cologne, Germany). Cleaved caspase 3 (Asp175) (D3E9) Rabbit mAb, Cleaved caspase 8 (Asp391) (18C8) Rabbit mAb, Anti-rabbit IgG (H+L), F(ab’)2 fragment (Alexa fluor^®^ 647 conjugate), Anti-rabbit IgG (H+L), F(ab’)2 fragment (Alexa fluor^®^ 488 conjugate) and Phosphorylated Histone H3 were purchased from Cell Signaling Technology (Danvers, MA, USA).

### 4.2. Plant Material and Extract Preparation

The aerial part of *A. nemorosa* was harvested from Piatra Neamţ, Neamţ county (April, 2014) and the identity was established by comparing with herbarium specimens from ‘Dimitrie Brandza’ Botanical Garden, Bucharest. Voucher specimens are available in the herbarium collection of the “Dimitrie Brandza” Botanical Garden, Bucharest (no. 405889) and at the Department of Botany and Cell Biology, “Carol Davila” University of Medicine and Pharmacy, Bucharest.

The aqueous extract of *A. nemorosa* was prepared by grounding approximately 10 g (mesh 14) and then extracting with 3 × 100 mL water under reflux. This was followed by concentration through rotary evaporation (RVO 004; Ignos, Prague, Czech Republic) and lyophilization at −55 °C (CoolSafe ScanVac 55; LaboGene, Lynge, Denmark), yielding 16.67% dry extract. The reproducibility of the extraction was evaluated on three batches obtained from the same plant material using IR spectra on a JASCO FT/IR-4200 spectrometer with an ATR PRO450-S accessory, on the spectral range of 4000–400 cm^−1^. For all experiments the plant extract was reconstituted in DMSO at a final concentration of 100 mg/mL and stored at −20 °C until use.

Total polyphenol content (TPC) and total flavonoid content (TFC) were determined according to the Folin-Ciocalteu method at λ = 750 nm and at λ = 429 nm using the aluminum chloride method [31]. The determinations were performed in triplicate using a UV-VIS spectrophotometer (Halo DB-20-220; Dynamica, Salzburg-Mayrwies, Austria). TPC and TFC were calculated using linear regression by interpolation on calibration curves made in the same conditions and expressed as the means ± SD of the experiments in milligram gallic acid equivalents (GAE) per gram of dry extract (DE) for TPC and in milligram quercetin equivalents (QE) per gram of DE for TFC. TPC was 23.98 mg GAE/g DE, whereas TFC was 1.12 mg QE/g DE.

### 4.3. Cell Culture Conditions

HeLa cells were routinely maintained in 10 cm culture dishes in RPMI 1640 culture medium supplemented with 10% fetal bovine serum and incubated at 37 °C in a humidified incubator with 5% CO_2_. Cell number and viability was determined by using a Luna^TM^ cell Counter (Logos Biosystems, Inc., Anyang, South Korea) after staining cells with trypan blue.

### 4.4. Experimental Imaging and Analysis

For all experiments, unless otherwise indicated, the ImageXpress Micro XLS Widefield High-Content Analysis System (Molecular Devices^®^, San Jose, CA, USA) was used to image cells. The images were analyzed using the applicable modules of the MetaXpress^®^ High-Content Image Acquisition & Analysis Software supplied by Molecular Devices^®^ (San Jose, CA, USA).

### 4.5. Cytotoxicity

HeLa cells were seeded in 96-well plates at densities of 5000 cells/well using 100 µL aliquots and left overnight to attach. For treatment, an additional 100 µL of the treatments at varying concentrations of *A. nemorosa* (0.1–300 µg/mL) and cisplatin (0.1–200 µM) were added. Treated cells were incubated at 37 °C in a humidified 5% CO_2_ incubator for 48 h. Treatment medium was removed and replaced with 100 µL phosphate buffered saline (PBS with Ca^2+^ and Mg^2+^) containing Hoechst 33,342 at a final concentration of 5 µg/mL. PI was added at final a concentration of 10 µg/mL using 10 µL per well of a 110 µg/mL stock and the cells were imaged.

### 4.6. Cell Cycle Analysis and Micronucleus Assay

HeLa cells were seeded in 96-well plates at a density of 5000 cells/well using 100 µL aliquots and left overnight to attach. The cells were treated with the IC_10_, IC_25_, IC_50_ and IC_75_ concentrations of *A. nemorosa* and cisplatin. Treated cells were incubated at 37 °C in a humidified 5% CO_2_ incubator for 24 and 48 h. Treatment medium was removed and cells were fixed using 4% formaldehyde for 15 min at room temperature prior to the addition of 100 µL NucRed^TM^ Live 647 stain (50 µL NucRed in 10mL PBS, according to manufacturer’s instructions). Cells were incubated for 15 min at room temperature prior to imaging.

### 4.7. Histone H3 Phosphorylation

HeLa cells were seeded and treated as described for cell cycle analysis. Staining was performed as described for cell cycle analysis, after which cells were permeabilized using 80% ice cold methanol at minus 20 °C for 10 min. Phospho-Histone H3 (Ser10) (D2C8) XP^®^ Rabbit mAb (Cell Signaling Technology, Danvers, MA, USA) was used to determine the presence of phosphorylated Histone H3. After permeabilization the cells were blocked using PBS containing 0.5% BSA and thereafter incubated with the primary antibody (1:250) for 1 h at 37 °C. Cells were washed and incubated with a conjugated secondary antibody (1:1000), anti-rabbit IgG (H+L), F(ab’)2 Fragment (Alexa Fluor^®^ 488 Conjugate) (Cell Signaling Technology, Danvers, Massachusetts, USA) for 30 min at 37 °C in the dark. After staining with the secondary antibody, the cells were washed once to eliminate unbound antibodies and then imaged.

### 4.8. Phosphatidylserine Translocation

The Annexin V-FITC/PI kit from MACS Miltenyi Biotec (Cologne, Germany) was used with modifications. HeLa cells were seeded and treated as described for cell cycle analysis. After 24 and 48 h, cells were stained by removing treatment medium and adding 50 μL aliquots of a mixture of Annexin V-FITC (50 μL) and Hoechst 33,342 (1 μL) in 5 mL PBS containing 250 μL of Binding Buffer (20×). Cells were incubated in the dark for 15 min at room temperature. PI (50 μL per well of 2 μg/mL stock) was added to the Annexin/Hoechst stain just before image acquisition.

### 4.9. Mitochondrial Membrane Potential (MMP) Analysis

HeLa cells were seeded and treated as described for cell cycle analysis. Cells were stained by removing treatment medium and adding 100 μL aliquots of a mixture of 0.05 mM tetramethylrhodamine ethyl ester (TMRE) (50 μL) and Hoechst 33,342 (2 μL) in 10 mL PBS. Cells were incubated in the dark for 30 min at 37 °C prior to image acquisition.

### 4.10. Caspase 8 and 3 Activation

HeLa cells were seeded and treated as described for cell cycle analysis analysis. Cells were treated for 24 and 48 h, after which they were fixed and permeabilized as described for histone H3 phosphorylation. Cleaved caspase 8 (Asp391) and cleaved caspase 3 (Asp175) (D3E9) rabbit monoclonal antibodies (Cell Signaling Technology) were used to determine the presence of activated caspase 8 and caspase 3, respectively. After permeabilization the cells were blocked using PBS containing 0.5% BSA and thereafter incubated with the antibodies separately (1:100 for caspase 8 and 1:200 for caspase 3) for 1 h at 37 °C. Cells were washed and incubated with a conjugated secondary antibody (1:500), anti-rabbit IgG (H+L), F(ab’)2 Fragment (Alexa Fluor^®^ 647 Conjugate) for 30 min at 37 °C in the dark. After staining with the secondary antibody, the cells were washed once to eliminate unbound antibodies and Hoechst 33,342 was used as a counterstain prior to imaging.

### 4.11. Reactive Oxygen Species (ROS) Production

HeLa cells were seeded and treated as described for cell cycle analysis. Cells were stained by removing treatment medium and adding 100 μL aliquots of a mixture of 2.5 mM CellRox orange (20 μL) and Hoechst 33,342 (2 μL) in 10 mL PBS. Cells were incubated in the dark for 30 min at 37 °C prior to image acquisition.

### 4.12. Autophagy Induction

HeLa cells were seeded and treated as described for cell cycle analysis. Cells were stained by removing treatment medium and adding 100 μL aliquots of a mixture of 50 nM Lysotracker red (0.5 μL) and Hoechst 33,342 (2 μL) in 10 mL PBS. Cells were incubated in the dark for 30 min at 37 °C prior to imaging.

### 4.13. Statistical Analysis

At least three experiments were completed for each cell line in which three different transfer numbers were used. Statistical analysis was performed by means of the student *t*-test for two samples assuming equal variance. Error bars represent the standard deviation of the mean (SD).

## 5. Conclusions

Taken together, our results demonstrate that the crude aqueous extract of *A. nemoros*a may have anti-cancer potential. This study has shown that upon treatment of HeLa cells with *A. nemorosa* apoptosis is induced. The mechanism of induction of apoptosis is accompanied by early M phase cell cycle arrest, induction of mitotic catastrophe, mitochondrial membrane depolarization, caspase activation and reactive oxygen species generation. Future studies will focus on the isolation and identification of active component(s) and to characterize the precise mechanism of action induced by *A. nemorosa*.

## Figures and Tables

**Table 1:** The inhibitory concentration values of Cisplatin and *A. nemorosa* on HeLa cells.

Treatment	IC_10_	IC_25_	IC_50_	IC_75_
*A. nemorosa* (µg/mL)	6.435 ± 0.785	11.44 ± 1.395	20.33 ± 2.480	36.14 ± 4.408
Cisplatin (µM)	0.068 ± 0.012	0.336 ± 0.060	1.675 ± 0.301	8.342 ± 1.499

**Table 2:** Changes in phosphorylated Histone H3 levels after 24 and 48 h of exposure to cisplatin and *A. nemorosa*.

Treatment	Cells Stained Positive for Phosphorylated Histone H3 (%)			
	*A. Nemorosa*		Cisplatin	
	24 h	48 h	24 h	48 h
control	12.28 ± 1.88	5.35 ± 0.52	12.28 ± 1.88	5.35 ± 0.52
IC_10_	11.54 ± 0.93	5.20 ± 0.68	13.01 ± 1.35	5.32 ± 0.99
IC_25_	11.17 ± 0.49	6.45 ± 0.77	11.95 ± 0.76	5.83 ± 1.23
IC_50_	11.50 ± 0.47	7.01 ± 0.69	7.90 ± 0.52 *	8.70 ± 1.42 *
IC_75_	41.31 ± 6.23 **	11.70 ± 1.77 **	6.04 ± 1.16 *	4.55 ± 1.47

Significance was determined using the two-tailed Student *t*-test: * *p* < 0.05 and ** *p* < 0.005 compared to control.

**Table 3:** Changes in cleaved caspase 8 levels in HeLa cells after 24 and 48 h of exposure to *A. nemorosa* extract and cisplatin.

Treatment	Cleaved Caspase 8 Mean Cell Integrated Intensity (% of control)			
	*A. Nemorosa*		Cisplatin	
	24 h	48 h	24 h	48 h
control	100.0 ± 1.15	100.0 ± 1.82	100.0 ± 1.15	100.0 ± 1.82
IC_10_	104.7 ± 4.47	102.7 ± 0.14	99.14 ± 1.23	102.9 ± 1.46
IC_25_	110.9 ± 12.6	102.7 ± 3.13	105.9 ± 2.66 *	108.2 ± 2.77 *
IC_50_	101.6 ± 4.98	107.9 ± 3.90 *	116.3 ± 0.21 **	151.8 ± 8.08 **
IC_75_	116.5 ± 17.5	112.8 ± 3.06 **	124.7 ± 2.24 **	167.2 ± 7.37 **

Significance was determined using the two-tailed Student *t*-test: * *p* < 0.05 and ** *p* < 0.005 compared to control.

**Table 4:** Changes in cleaved caspase 3 levels in HeLa cells after 24 and 48 h of exposure to *A. nemorosa* extract and cisplatin.

	Cleaved Caspase 3 Mean Cell Integrated Intensity (% of control)			
Treatment	*A. Nemorosa*		Cisplatin	
	24 h	48 h	24 h	48 h
control	100 ± 4.86	100 ± 1.17	100.0 ± 4.86	100.0 ± 1.17
IC_10_	101.5 ± 4.65	100.2 ± 2.46	103.5 ± 5.20	105.1 ± 1.94 *
IC_25_	102.8 ± 5.28	104.4 ± 0.19 **	110.6 ± 5.94	111.2 ± 3.29 *
IC_50_	103.4 ± 5.64	108.4 ± 2.65 **	123.2 ± 5.28 *	155.7 ± 7.82 **
IC_75_	106.1 ± 4.95	126.7 ± 4.31 **	129.1 ± 6.23 **	174.0 ± 8.58 **

Significance was determined using the two-tailed Student *t*-test: * *p* < 0.05 and ** *p* < 0.005 compared to control.

## Supplementary Materials

Supplementary materials can be found at https://www.mdpi.com/1422-0067/20/5/1217/s1.

## Abbreviations

AIF	apoptosis inducing factor
MMP	mitochondrial membrane potential
PI	propidium iodide
PS	phosphatidylserine
ROS	reactive oxygen species
SD	standard deviation of the mean
TMRE	tetramethylrhodamine ethyl ester
