# Supplementary material for: In Vitro Anti-proliferative Activity and Mechanism of Action of Anemone nemorosa
Source: Int J Mol Sci. 2019 Mar 11;20(5):1217. doi: 10.3390/ijms20051217 (PMC6429291; doi:10.3390/ijms20051217)
Supplement: Supplementary file 1 [file ijms-20-01217-s001.pdf]

International Journal of
Molecular Sciences

Supplementary Materials

In Vitro Anti-Cancer Activity and Mechanism of Action of Anemone Nemorosa

Bresler Swanepoel [1], Luanne Venables [1], Octavian Tudorel Olaru [2], George Mihai Nitulescu [2]* and Maryna van de Venter [1]

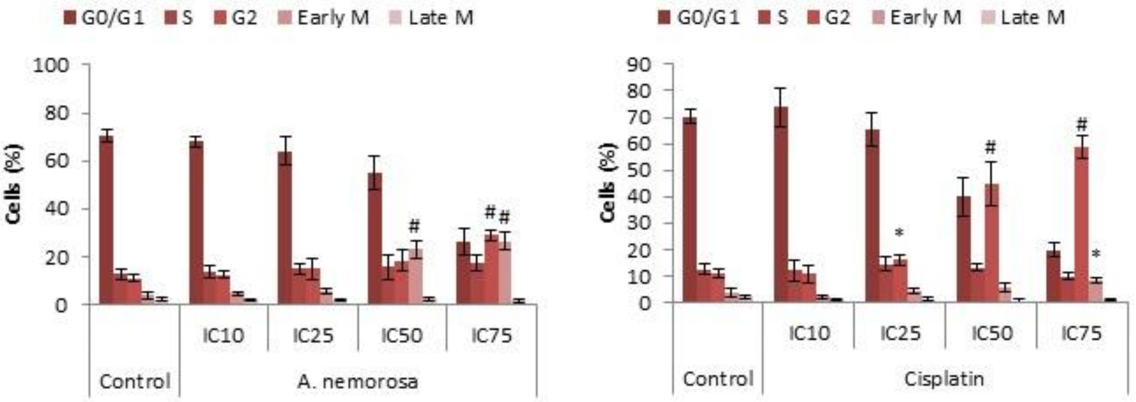

(**a**) (**b**)

Figure S1. Cell Cycle Analysis of HeLa cells after 48 hours of treatment with *A. nemorosa* (a) and cisplatin (b). Cell cycle analysis was determined by the NucRed Live 647 staining method. Error bars indicate SD of four replicate values of three individual experiments. Significance was determined using the two-tailed Student t-test: *p<0.05 and #p<0.005 compared to control.

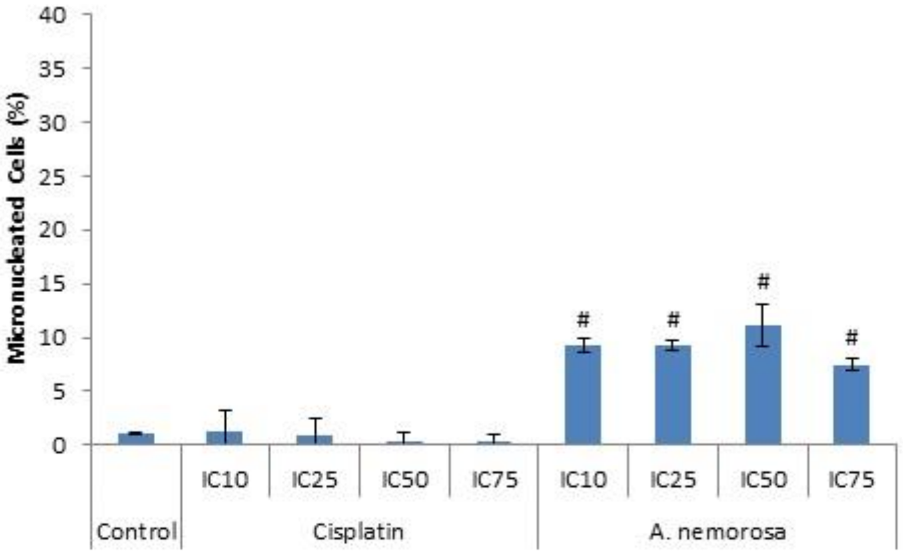

Figure S2. Assessment of genotoxicity in HeLa cells after 24 hours using the NucRed Live 647 staining method. Error bars indicate SD of four replicate values of three individual experiments. Significance was determined using the two-tailed Student t-test: #p<0.005 compared to control.

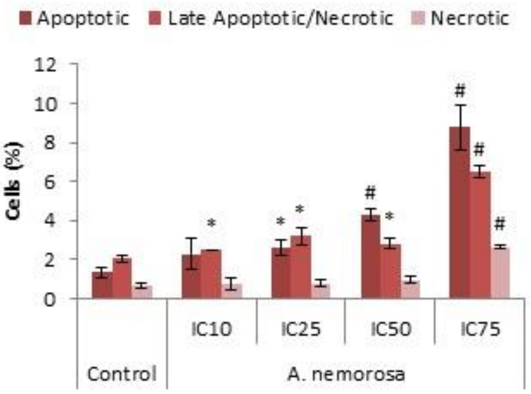

(**a**)

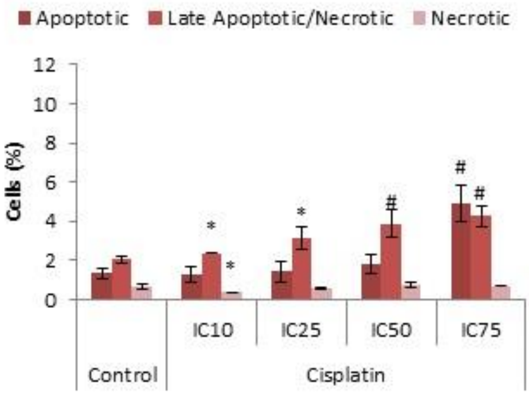

(**b**)

Figure S3. Analysis of PS translocation in HeLa cells using Annexin V-FITC and PI dual staining after 24 hours of treatment with *A. nemorosa* (a) and cisplatin (b). Results displayed as percentage positively stained cells. Error bars indicate SD of four replicate values of three individual experiments. Significance was determined using the two-tailed Student t-test: *p<0.05 and #p<0.005 compared to control.

© 2019 by the authors. Licensee MDPI, Basel, Switzerland. This article is an open access article distributed under the terms and conditions of the Creative Commons Attribution (CC BY) license (http://creativecommons.org/licenses/by/4.0/).
